# HMP-1/α-catenin promotes junctional mechanical integrity during morphogenesis

**DOI:** 10.1371/journal.pone.0193279

**Published:** 2018-02-21

**Authors:** Thanh Thi Kim Vuong-Brender, Arthur Boutillon, David Rodriguez, Vincent Lavilley, Michel Labouesse

**Affiliations:** 1 Sorbonne Universités, UPMC Université Paris 06, CNRS, Laboratoire de Biologie du Développement—Institut de Biologie Paris Seine (LBD—IBPS), Paris, France; 2 Development and Stem Cells Program, IGBMC, CNRS (UMR7104), INSERM (U964), Université de Strasbourg, 1 rue Laurent Fries, llkirch, France; University of Illinois at Chicago, UNITED STATES

## Abstract

Adherens junctions (AJs) are key structures regulating tissue integrity and maintaining adhesion between cells. During morphogenesis, junctional proteins cooperate closely with the actomyosin network to drive cell movement and shape changes. How the junctions integrate the mechanical forces in space and in time during an *in vivo* morphogenetic event is still largely unknown, due to a lack of quantitative data. To address this issue, we inserted a functional Fluorescence Resonance Energy Transfer (FRET)-based force biosensor within HMP-1/α-catenin of *Caenorhabditis elegans*. We find that the tension exerted on HMP-1 has a cell-specific distribution, is actomyosin-dependent, but is regulated differently from the tension on the actin cortex during embryonic elongation. By using time-lapse analysis of mutants and tissue-specific rescue experiments, we confirm the role of VAB-9/Claudin as an actin bundle anchor. Nevertheless, the tension exerted on HMP-1 did not increase in the absence of VAB-9/Claudin, suggesting that HMP-1 activity is not upregulated to compensate for loss of VAB-9. Our data indicate that HMP-1 does not modulate HMR-1/E-cadherin turnover, is required to recruit junctional actin but not stress fiber-like actin bundles. Altogether, our data suggest that HMP-1/α-catenin acts to promote the mechanical integrity of adherens junctions.

## Introduction

Adherens junctions (AJs) are conserved structures essential to maintain tissue architecture and cell-to-cell communication. They have been the subject of extensive investigation since their discovery many decades ago [1]. Studies in both cell culture and living embryos have identified the core components of AJs, the cadherin-catenin complex (CCC), and their importance in cell physiology, organism development and disease [2, 3]. E-cadherin is a Ca^2+^ binding transmembrane protein that forms *trans*-homodimers, bridging the plasma membranes of two adjacent epithelial cells [4]. E-cadherin contains a cytoplasmic tail that binds β-catenin, which in turn binds to α-catenin. Finally, α-catenin mediates the dynamic link between the CCC and the actin cytoskeleton, allowing mechanical coupling between the inside of the cell and its borders [5, 6]. This connection is crucial for all morphogenetic processes involving epithelia, such as *Caenorhabditis elegans* ventral enclosure [7, 8], apical constriction during *Drosophila* mesoderm invagination [9] or germband extension [10].

*C*. *elegans* is a versatile system frequently used to study AJs *in vivo*, in a complementary manner to studies in mammalian cell culture [11]. Mutations affecting *C*. *elegans* CCC components exhibit severe body morphology defects during embryonic development, showing their importance for embryonic morphogenesis [7]. *C*. *elegans* contains only one classic cadherin which is named HMR-1. In embryos lacking HMR-1/E-cadherin, ventral epidermal cells fail to establish contacts, leaving the head uncovered [7, 8]. Depletion of maternal and zygotic expression of HMP-1/α-catenin leads to the same phenotype as zygotic loss of HMR-1 [7, 8]. In particular leader cells fail to maintain contacts if HMP-1 is not recruited to nascent contacts [8], indicating that a functional adhesion by E-cadherin must be reinforced by the connection with the cytoskeleton. The importance of the link between HMP-1 and F-actin during embryonic elongation was demonstrated using mutants in the actin binding domain of HMP-1 [12]. These mutants show a detachment of actin bundles from cell junctions in the dorso-ventral epidermis, leading to inhibition of elongation [7, 12]. In contrast to mammalian α-catenin which makes homodimers [13], HMP-1 exists in a monomeric form in the cytosol [14]. Furthermore, in contrast to mammalian cells which recruit vinculin when α-catenin has been extended under force [15, 16], this process does not occur in *C*. *elegans* epithelial cells as they do not express vinculin [17]. Other actin-binding proteins localizing at the junctions, such as ZOO-1 (Zonula Occludens subfamily) and AFD-1/Afadin, act together with HMP-1 to strengthen adherens junctions [18–20]. ZOO-1 acts downstream of VAB-9/Claudin [18], whereas AFD-1/Afadin localization to the junctions depends on a pathway involving SAX-7/L1CAM and MAGI-1 [19].

**Fig 1:**
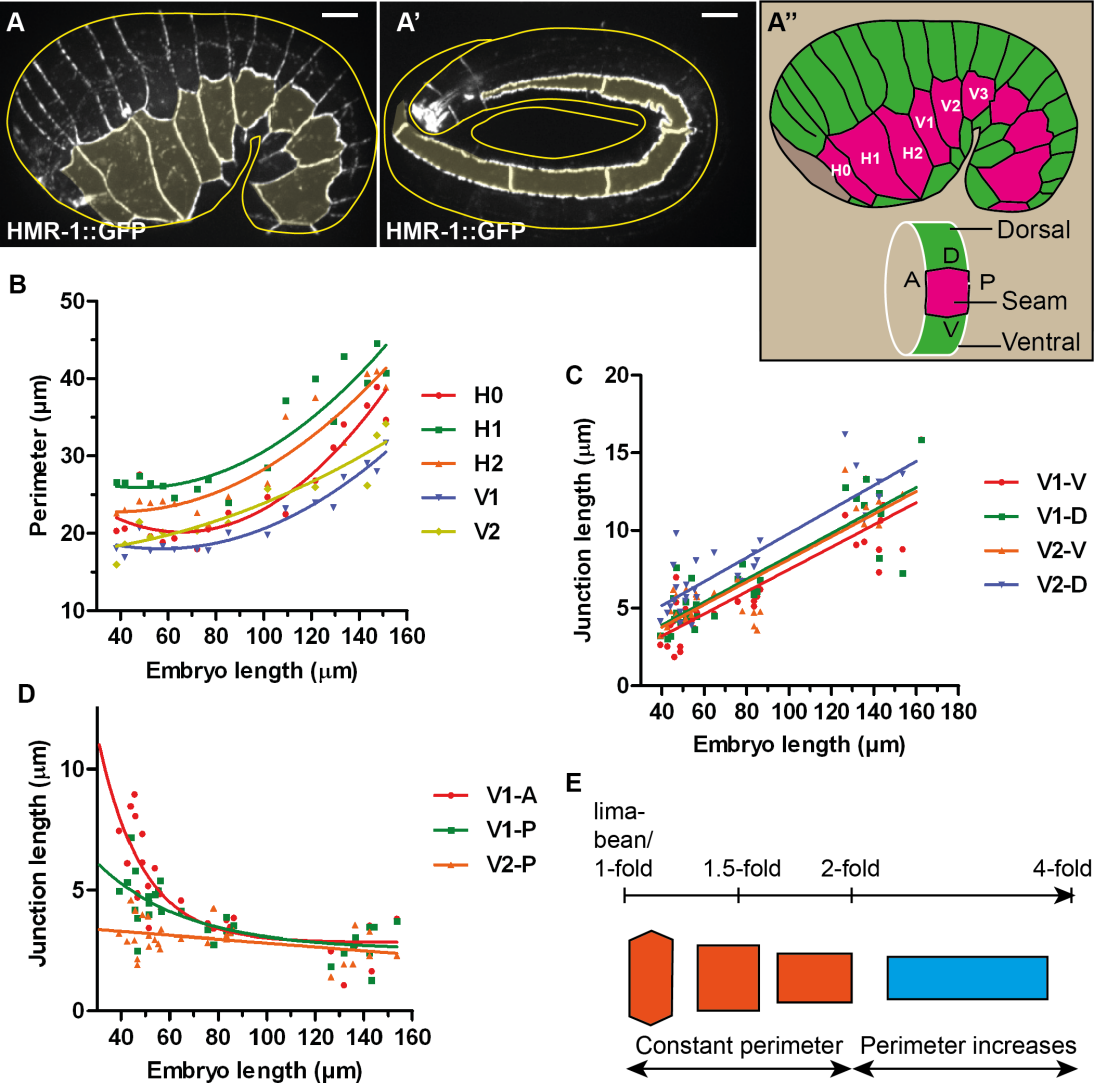
Two distinct phases of junction remodelling. (A-A’) Seam cell adherens junctions of an early (A) and late elongation (A’) *C*. *elegans* embryo marked by the CRISPR HMR-1/E-cadherin GFP-fusion reporter. Seam cells are shaded in yellow. Yellow lines show approximately the embryo contour. Note that in A’, the embryo is like a tube folded within the eggshell, only 5 μm on top of the embryo was projected and the five first seam cells are visible. Scale bar, 5 μm. (A”) Schematic representation of seam (magenta) and dorsal-ventral epidermis (green) based on the embryo in (A). The position of the seam cells from H0 to V3 is shown; anterior to the left, dorsal up. A, P, D and V represent the anterior, posterior, dorsal and ventral junctions of the cell examined. (B) Measurements of seam cell perimeter showing slight changes up to the 2-fold stage (around 80–90 μm in embryo length) then a clear increase after this stage. Curves are quadratic fit to show the variation trend. (C) Length measurements of V1 and V2 dorsal (D) and ventral (V) junctions showing a linear increase in dorso-ventral junction length. Solid lines show linear fit. (D) Length measurements of seam-seam junctions showing a variable decrease rate until they all reach a similar junction length at the 2-fold stage. The seam-seam junction length decreased only slightly beyond that stage. Curves are single exponential decay fit to show the variation trend. (E) Scheme showing two phases of seam cell shape changes: with a constant perimeter from lima-bean to 2-fold, then an increasing perimeter due to elongation of only dorso-ventral junctions in the antero-posterior axis.

The importance of the HMP-1/α-catenin linkage to actin suggests that it is a major transmitter/transducer of actomyosin contractility, ensuring tension continuity across junctions. However, force measurements are lacking to show how actomyosin tension on AJs is distributed and regulated. It is also unclear whether this tension gives cues to junctional proteins to remodel according to the morphological changes of the embryo. To address these issues, we assessed the tension exerted on HMP-1 using a novel force tension biosensor based on Fluorescence Resonance Energy Transfer (FRET); we inserted the biosensor between the ß-catenin-binding and actin-binding domains of HMP-1/α-catenin. We measured the tension distribution and evolution during *C*. *elegans* embryonic elongation, when the embryo increases four fold in length along the head-to-tail axis (the final stage is referred to as the 4-fold stage). Epidermal actomyosin contractility and muscle contractions are the major drivers of this process [21–23]. During elongation, lengthening along the antero-posterior and shortening along the transverse direction of epidermal cell junctions are prominent [7, 21] (Fig 1A and 1A’), suggesting that junctions must be remodelled to accommodate these changes. We found that tension on HMP-1 has local variation and is not directly correlated with cortical actomyosin tension. On the other hand, our characterization of actin and HMR-1/E-cadherin distribution in *hmp-1* mutants prompted us to significantly revise the proposed role for HMP-1/α-catenin at junctions.

## Results

### *C*. *elegans* epidermal junctions elongate in two phases

We first quantified the dynamics of cell shape changes during elongation. We used fixed embryos immuno-stained with the junctional marker MH27 [24]. We measured the perimeter and the junction length along the antero-posterior and the circumferential axis of different seam cells (Fig 1A”), and plotted these parameters as a function of the embryo length. For a given cell, we call A, P, D and V respectively the anterior, posterior, dorsal and ventral junctions of the cell (Fig 1A”). For example H1-P, which is also H2-A, is the seam-seam junction between H1 and H2. As shown in Fig 1B for five seam cells (H0 to V2), their perimeter changed only slightly until the embryo reached the two-fold stage and then increased regularly. Meanwhile, the D and V junctions increased almost linearly with the embryo length (Fig 1C). By contrast, the A and P junctions showed a fast decrease until the 2-fold stage, then a slight decrease thereafter. The constant then increasing perimeter before and after the 2-fold stage (Fig 1B) can be explained by the opposite variation in length of D/V and A/P junctions up to the 2-fold stage, then the elongation of D/V junctions without notable shrinking of A/P junctions.

Hence, the seam junctions remodelled following two distinct periods (Fig 1E), one with a constant perimeter until the 2-fold stage, and one with an increasing perimeter after this stage. This observation fits with genetic data suggesting that embryonic elongation involves distinct pathways before and after the two-fold stage. Indeed, the *C*. *elegans* ROCK homolog LET-502 is essential until the two-fold stage [25], at which point muscle tension promotes epidermal cell shape changes through a mechanotransduction pathway [26]. The different behaviour of seam-seam and seam-dorso/ventral junctions suggests that these junctions must remodel differently.

### A FRET-based force sensor shows a decrease of tension exerted on HMP-1/α-catenin from the 1.3-fold to the 1.5-fold stages

**Fig 2:**
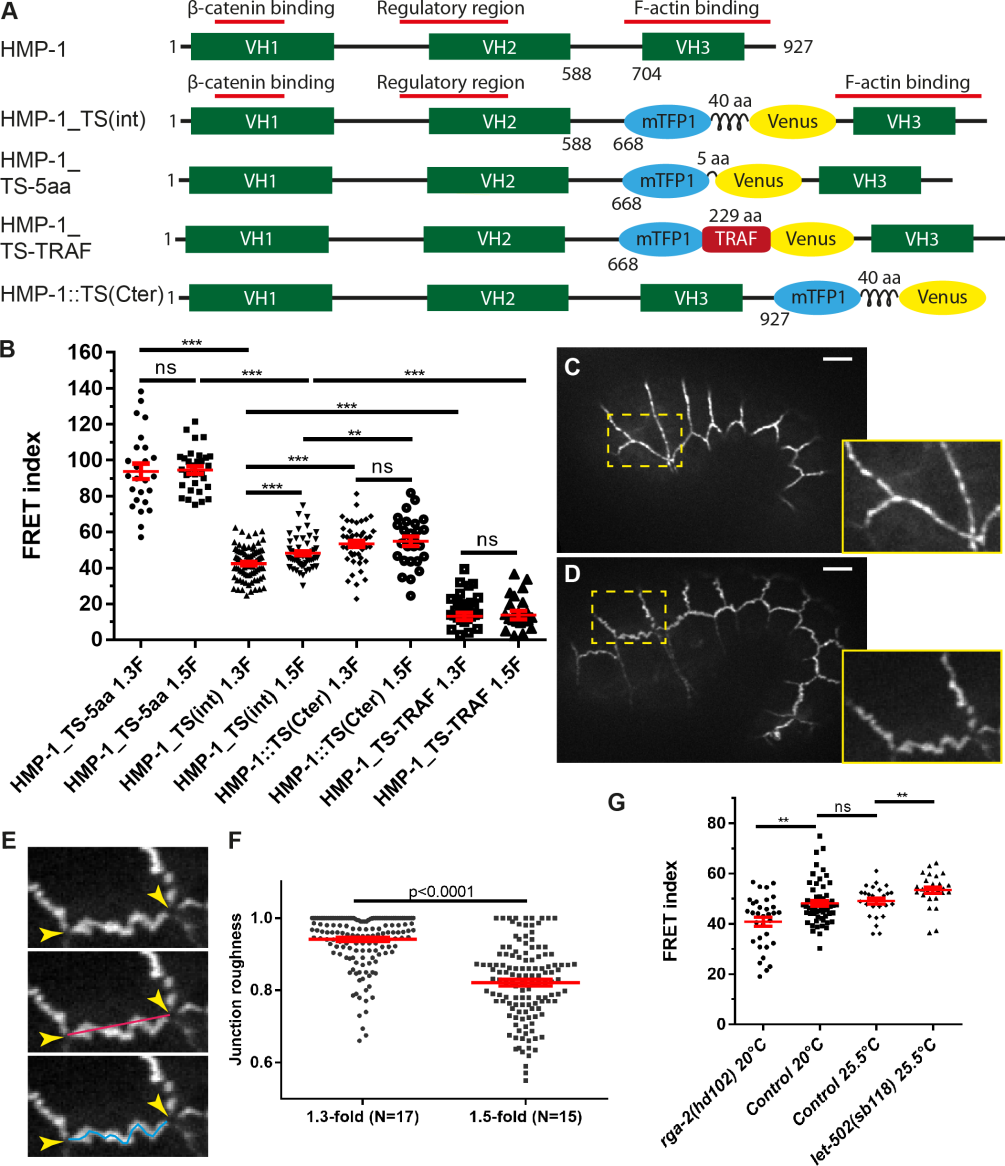
A HMP-1/α-catenin tension sensor measures actomyosin-dependent forces on the cadherin-catenin complex. (A) Scheme showing the position and structure of the different constructs used to measure forces on HMP-1 after CRISPR knock-in. The number along the HMP-1 protein denotes the residue number; the length of the linker between mTFP1 and Venus is also shown in amino acid (aa). VH: vinculin homology domain. (B) FRET index of the different CRISPR constructs in (A) measured at the 1.3-fold (1.3F) and 1.5-fold (1.5F) stages. The number of embryos used for FRET is shown in S2 Table. Note that for HMP-1_TS-TRAF, the FRET index (FRET/donor fluorescence) calculated in heterozygote embryos should report the same value as in homozygote embryos. (C-D) Fluorescence pictures of an embryo at the 1.3-fold (C) and 1.5-fold (D) stages showing more wrinkled junctions at the later stage. Lower right panels show a close-up view of the area with a dashed rectangle. (E) Scheme showing that the junctional roughness was measured as the ratio between the shortest line (red line) joining all vertices (arrowheads) and the actual length of the junction (blue line). The straighter the junction, the closer the roughness is to 1. (F) Roughness of junctions at the 1.3-fold and the 1.5-fold stages. N, number of embryos. (G) Comparison of the FRET index (measured using HMP-1_TS(int) construct) between the control, *rga-2(hd102)/RhoGAP* and *let-502(sb118)/Rho kinase* mutant embryos. *let-502(sb118)* is a thermosensitive mutant and was measured at the restrictive temperature 25.5°C. p-values of Mann-Whitney test are reported. ns, not significant; *, p<0.05; **, p<0.01; ***, p<0.001. Red lines in (B,F,G) show mean and s.e.m (standard error of the mean). Scale bar, 5 μm.

To assess the tension on the adherens junctions, we inserted the TSMod FRET-based force sensor module [27–29] in HMP-1/α-catenin. We chose to position the TSMod within HMP-1 before its actin-binding VH3 domain, as originally done for vinculin to measure forces exerted on focal adhesions [27], since α-catenin and vinculin share the same general structure with 28% sequence identity (Fig 2A). The force sensor contains the donor-acceptor fluorescence molecule pair, mTFP1 and Venus, separated by a spider silk repeats of 40 amino acid (aa) long. The energy transfer between the two molecules decreases with increasing extension forces applied on the module [27]. We used the CRISPR/Cas9 method to insert this module as well as all control constructs (Fig 2A, HMP-1_TS(int)). To know the dynamic range of this sensor in our system, we built constitutively high- and constitutively low-FRET lines by replacing the 40 aa linker with a short 5-residue linker and the 229 residue-long TRAF domain from the Tumor Necrosis Factor receptor-associated factor [28], respectively (Fig 2A). To compare with a situation in which no force would be exerted on the sensor, we fused the sensor at the C-terminus of HMP-1 (HMP-1::TS(Cter)). All the CRISPR lines (except the HMP-TS_TRAF) could be kept as homozygotes and were healthy (S1 Table), showing that the TSMod did not alter protein function. We focused on early elongation stages, when the absence of muscle contractions allowed the fluorescence measurements.

To compare the different backgrounds, we calculated a FRET index (see Materials and Methods) that correlates with the energy transfer and thus reports on changes in tension. Fig 2B shows the FRET index measured at the 1.3-fold and 1.5-fold stages for the whole embryo. We first validated the HMP-1_TS(int) line by comparing its FRET index with that of control lines. The constitutively high- and low-FRET lines (HMP-1_TS-5aa and HMP-1_TS-TRAF respectively) showed a significantly higher and lower FRET index than the internal TS inserted in HMP-1 (HMP-1_TS(int)). Likewise, the FRET index of the HMP-1_TS(int) construct, which measured the force applied though HMP-1 to the adherens junctions, was significant smaller than the values measured with the HMP-1::TS(Cter) construct, which reflects the situation without force exerted. Interestingly, we observed a lower FRET index for HMP-1_TS(int) at the 1.3-fold stage compared to 1.5-fold stage, indicating that the forces applied on HMP-1 decreased between both stages. This force difference correlates with the difference in junction morphology, since junctions appeared more stretched at the 1.3-fold stage compared to the 1.5-fold stage, as shown by the measurement of junction roughness (Fig 2C–2F). As expected for the HMP-1::TS(Cter) control, no significant difference in FRET index was observed between these two stages.

To determine if the HMP-1_TS(int) construct effectively measured actomyosin tension, we compared the FRET index in wild-type embryos and in mutants that up-regulate or down-regulate myosin II activity. RGA-2/RhoGAP has been shown to inhibit myosin II in dorso-ventral cells; its depletion induces junction anomalies and embryo rupture [25]. Conversely, LET-502/Rho Kinase increases myosin II activity through phosphorylation of myosin light chain MLC-4 and promotes elongation [30]. In *rga-2*(*hd102)* and thermosensitive *let-502(sb118)* mutant raised at restrictive temperature (25.5°C), we found a significant decrease and increase of FRET index, respectively (Fig 2G), consistent with an increase and decrease of forces exerted on the junctions, respectively. Thus, the HMP-1_TS(int) sensor reports on actomyosin-dependent tension. Altogether, the comparison between the internal construct with control constructs, as well as with hyper- and hypo-tension situations, shows that the HMP-1_TS(int) can measure actomyosin tension exerted on the adherens junctions through HMP-1.

In summary, by developing a TSMod FRET sensor within HMP-1/α-catenin we could confirm that HMP-1/α-catenin is sensitive to tension and observed a decrease in actomyosin-dependent tension on HMP-1 from the 1.3-fold to the 1.5-fold stages.

### Loss of VAB-9/Claudin does not affect the tension exerted on HMP-1/α-catenin

**Fig 3:**
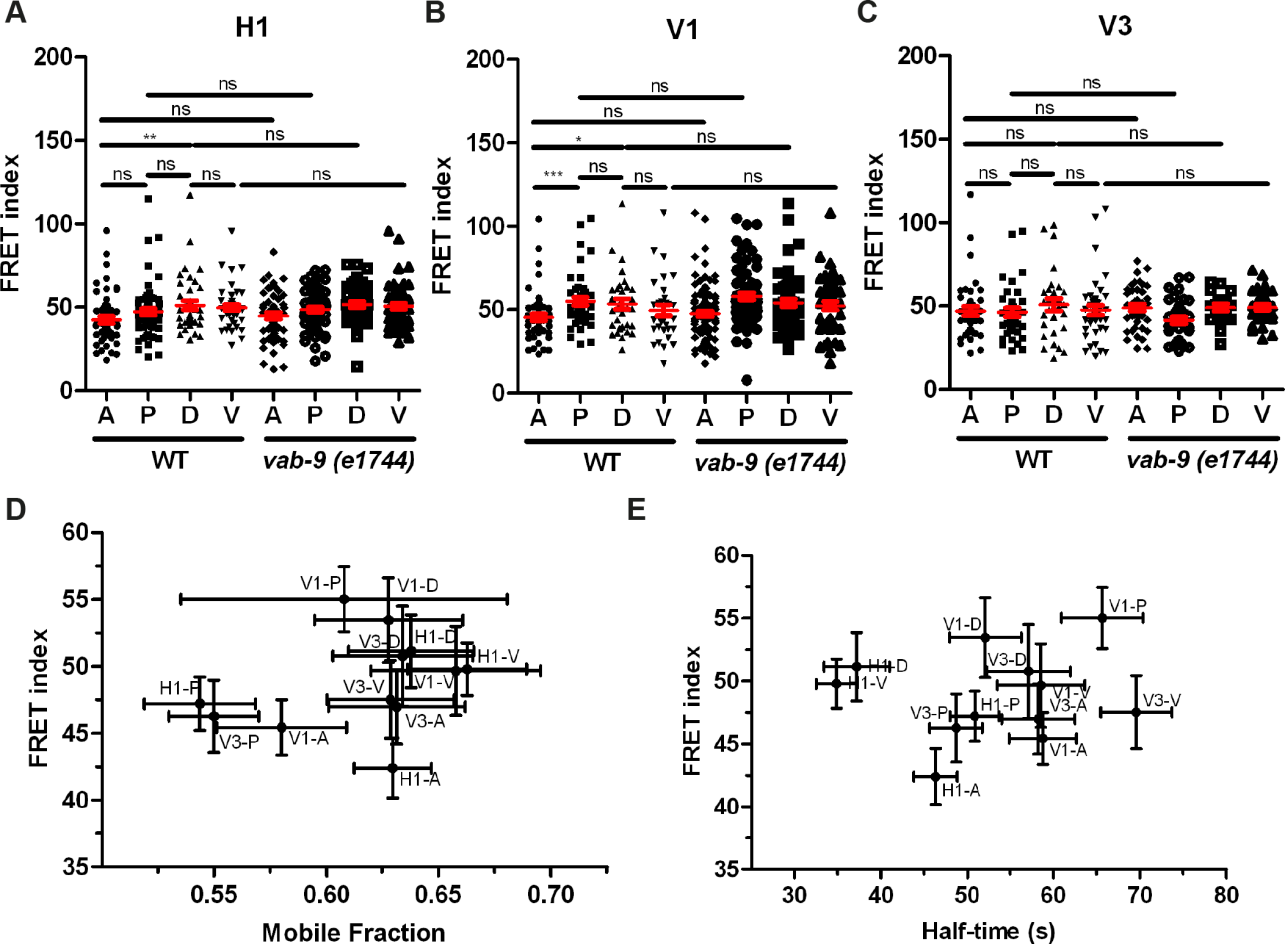
Tension on HMP-1/α-catenin is not significantly affected by loss of VAB-9/Claudin and is not correlated with HMR-1/E-cadherin turnover rate. (A-C) FRET index measured (using HMP-1_TS(int)) for anterior (A), posterior (P), dorsal (D), and ventral (V) junctions of the seam cells H1 (A), V1 (B), and V3 (C) in wild-type and *vab-9(e1744)* mutant embryos. Mean and s.e.m are shown in red. p-values of Mann-Whitney test are reported. ns, not significant; *, p<0.05; **, p<0.01; ***, p<0.001. The number of embryos used for FRET is shown in S3 Table. (D and E) FRET index for different wild-type seam cell junctions (summary from **A**-**C**) plotted versus the mobile fraction (D) or half-time (E) of HMR-1 turnover (Parts C-F in S1 Fig) showing the absence of correlation.

To map the tension distribution on individual junctions, we measured the FRET index (using HMP-1_TS(int)) for each junction of the three seam cells H1, V1 and V3 at the 1.5-fold stage. The FRET index showed a cell-specific pattern (Fig 3A–3C) with no difference between dorsal and ventral junctions but variation among anterior and posterior (seam-seam) junctions. We found that the tension on the anterior junction was higher (corresponding to lower FRET index) compared to the dorsal junction in H1 and V1 but not in V3 (Fig 3A–3C). Based on the FRET index, the tension for the anterior junction was higher than that of the posterior junction only in V1, but no difference was observed in H1 and V3 (Fig 3A–3C).

**Fig 4:**
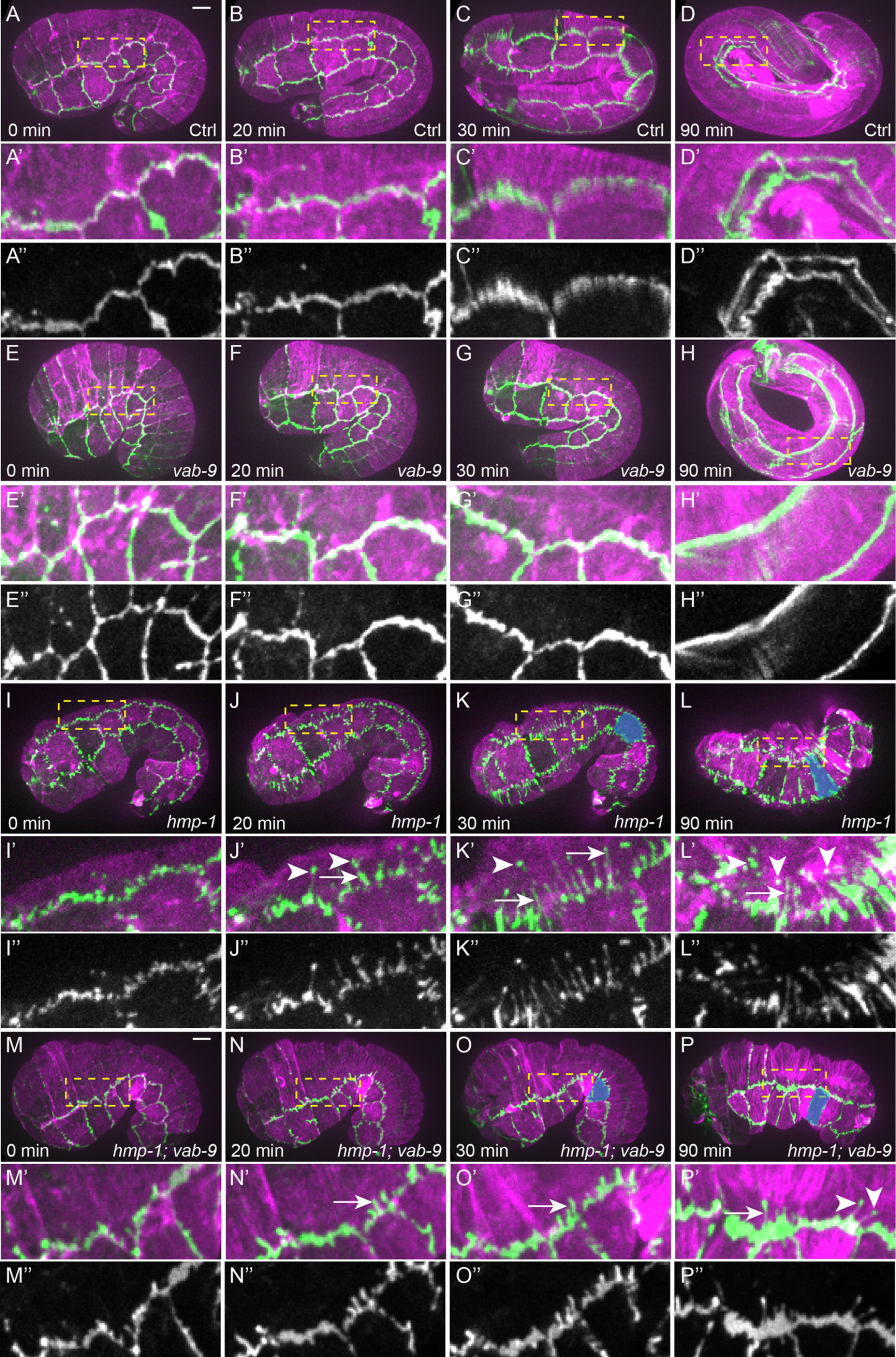
HMP-1 maintains the junction mechanical integrity whereas VAB-9 anchors actin bundles at seam-dorsal/ventral junctions. (A-P) Time-lapse sequence of embryos carrying the CRISPR construct HMR-1::GFP (green) and an integrated actin reporter (ABD::mCherry, magenta) in control (A-D), *vab-9(e1744)* (E-H), *hmp-1(zu278)* (I-L) and *hmp-1(zu278); vab-9(e1744)* (M-P) embryos. (A’-P’) Close-up images of the dashed rectangle in (A-P). (A”-P”) Single channel HMR-1::GFP of images (A’-P’). Arrows show the extension of HMR-1::GFP at seam-dorsal junctions in the dorsal direction; arrowheads show spots of HMR-1::GFP in the dorsal epidermal cell cytoplasm, some of which are still connected to where the bulk of the junctions through thin HMR-1-labelled extensions. Note the change of the V4 cell shape in late *hmp-1(zu278)* embryos compared to earlier stage (blue shading, K-L, O-P). Scale bar, 5 μm.

VAB-9/Claudin and HMP-1/α-catenin act in two sub-complexes linking actin to junctions; their depletion shows the most severe body morphology defects among the proteins associated with adherens junctions [7, 31]. However, *vab-9* mutants showed almost no defects during early elongation (Fig 4), only some body morphological anomalies are observed at the first larval stage [31]. To test if an increase in actomyosin-dependent tension on HMP-1 compensates the loss of VAB-9, we compared the FRET index in control embryos with *vab-9(e1744)* mutant, which is a nonsense mutation at codon 166 [31]. We found no significant difference between the FRET index between these two genotypes for all the junctions of H1, V1 or V3 (Fig 3A–3C), suggesting that loss of VAB-9 does not reallocate the tension onto HMP-1.

### HMR-1/E-cadherin turnover does not depend on the tension exerted on HMP-1/α-catenin

A recent study based on cultured cells has suggested that adherens junctions can sense forces exerted on them and modify the turnover of E-cadherin accordingly [32]. In a similar manner, the tension created by actomyosin to drive embryonic morphogenesis might promote junction remodelling through its associated actin-binding proteins. To assess the dynamics of junction remodelling, we used FRAP to measure the turnover of GFP-labelled endogenous HMR-1 [33]. We photo-bleached the A, P, D, and V junctions of the seam cells H1, V1, and V3 at the 1.5-fold stage, then derived the HMR-1 mobile fraction and half-time of recovery (Parts A-B in S1 Fig). We found some variation of mobile fraction among seam-seam cell junctions but no difference among seam-dorso/ventral junctions (Parts C-D in S1 Fig), whereas the half-time shows a cell-specific pattern (Parts E-F in S1 Fig). Plotting FRET index versus mobile fraction or half-time of recovery (Fig 3D and 3E), we found no correlation between them, suggesting that the turnover of HMR-1/E-cadherin does not depend on the actomyosin tension exerted on HMP-1.

To examine whether loss of junctional proteins affect the turnover of HMR-1, we measured the recovery of HMR-1::GFP in *vab-9(e1744)* and *hmp-1(zu278)* mutants. *vab-9(e1744)* mutation either did not change the mobile fraction or only mildly increased it at some seam-seam junctions (Parts C-D in S1 Fig). *zu278* is a C to T substitution resulting in a stop at the beginning of the actin binding domain (Q795Stop) [14] and thus the HMP-1 protein expressed should not be able to bind actin. In *hmp-1(zu278)* mutants, the mobile fraction decreased at some junctions, in particular in the seam cell H1 (Parts C-D in S1 Fig). Altogether, the differences in the half-time recovery between wild-type and *vab-9* or *hmp-1* mutant embryos were either quite small or not significant (Parts E-F in S1 Fig), and they did not affect all junctions, indicating that VAB-9 and HMP-1 marginally affect this parameter.

In summary, we found that actomyosin tension on the CCC acting through HMP-1 does not significantly impact on HMR-1/E-cadherin turnover, and that the junction-associated proteins HMP-1 and VAB-9 do not play a key role in regulating HMR-1 turnover.

### Loss of HMP-1 leads to local force unbalance at seam-dorso/ventral junctions

It was originally suggested [7] that HMP-1 anchors circumferential actin bundles to HMR-1 in the dorso-ventral epidermis since the bundles appear to detach from cell junctions in *hmp-1* mutants at the end of elongation. Our recent findings demonstrating that the tension exerted on cortical actin increases as the embryo elongates are consistent with this view [34]. However, the two set of observations seem to contradict the results reported above that the tension exerted on junctional HMP-1 decreases from the 1.3-fold to the 1.5-fold stage. To try and reconcile these data, we decided to revisit the characterization of actin defects in *hmp-1* mutants over time. We made time-lapse fluorescence movies of *hmp-1(zu278)* embryos expressing both a red actin marker (mCherry-labeled actin binding domain: ABD::mCherry) and green endogenous HMR-1/E-cadherin (HMR-1::GFP). Compared to controls, *hmp-1(zu278)* embryos showed fragmented junctions beyond the 1.5-fold stage (S2 Fig, Fig 4A, 4B”, 4I and 4J”). Moreover, they exhibited filamentous extensions perpendicular to the junctions into dorso/ventral (DV) epidermal cells. In these cells, dots of HMR-1 were present, some of which were still associated with the extension tip emanating from junctions. This phenotype is reminiscent of the defects seen in embryos depleted of UNC-94/Tropomodulin in a weak *hmp-1* mutant (*fe4*), however we did not see a ripping apart of the borders between seam and DV cells as observed in this background [35]. In parallel, the *hmp-1(zu278)* seam-seam junctions which had started to shorten before the 1.5-fold stage elongated back in the circumferential direction (Fig 4K and 4L, blue shading). Hence, we suggest that HMR-1 is still connected to actin filaments in the DV cells of *hmp-1* mutants, and that the HMR-1 dots visible in DV cells are pulled away from the seam-dorso/ventral cell borders due to higher tension from the bundles. This displacement of actin bundles towards the DV cells in the absence of HMP-1 suggests that mechanical integrity of the junction is compromised, and that the force exerted on one side of the seam-dorso/ventral epidermal junction is not balanced on the other side at the position where actin bundles contact the junctions (otherwise HMR-1 with actin bundle position would remain unchanged). This local force unbalance seemed to be due to a failure of the seam/DV cell junction to resist or to distribute forces evenly. Our data shows that HMP-1 is not essential to link actin bundles to HMR-1.

The previously observed HMR-1 displacement together with actin within the DV cell cytoplasm predicts that disconnecting actin from HMR-1 may rescue these defects. We chose to examine *hmp-1(zu278); vab-9(e1744)* double mutants, since VAB-9 has been proposed to anchor actin bundles to HMR-1 via ZOO-1 [18, 31]. In embryos defective for both HMP-1 and VAB-9, we found less fragmented junctions (compare Fig 4I and 4J” with Fig 4M and 4N”). Specifically, the extension of HMR-1 within the DV cell cytoplasm and the number of HMR-1 dots were reduced compared to *hmp-1(zu278)* mutants (compare Fig 4K–4K” with Fig 4O–4O”, 13.8±2.4 extensions in to dorsal cells in *hmp-1(zu278)* mutant, N = 5, compared to 5.8±1.6 extensions in *hmp-1(zu278); vab-9(e1744)* double mutant, N = 7, Mann-Whitney test p = 0.001). *vab-9(e1744)* mutant did not show any notable defects compared to wild-type embryos (Fig 4E–4H”). Note that we only focused on the defects during early elongation (<2-fold stage) since muscle contractions at later stages did not allow a good visualization *of* junctional abnormalities by time-lapse imaging. There were still HMR-1 extensions in the *hmp-1(zu278); vab-9(e1744)* double mutant, indicating that the truncated VAB-9 mutant protein may still allow some residual anchoring activity or that other proteins act redundantly with VAB-9 to mediate bundle anchoring to HMR-1. The *hmp-1; vab-9* double mutants still had abnormally extended seam cells along the circumference at late elongation (Fig 4O and 4P, blue shading), suggesting that there was still pulling of the seam cells from dorso/ventral cells even with compromised junctions.

In summary, our results support the hypothesis that the actin bundle retraction in *hmp-1* mutant is due to local force unbalance at the seam-dorso/ventral cell borders. They confirm the role of VAB-9 as an actin bundle anchor at the junctions, but suggest that HMP-1/α-catenin preserves the junctional mechanical integrity rather than anchors actin bundles from the DV cells to HMR-1.

### Restoring HMP-1 either in seam or DV cells rescues the Humpback phenotype and embryonic lethality

**Fig 5:**
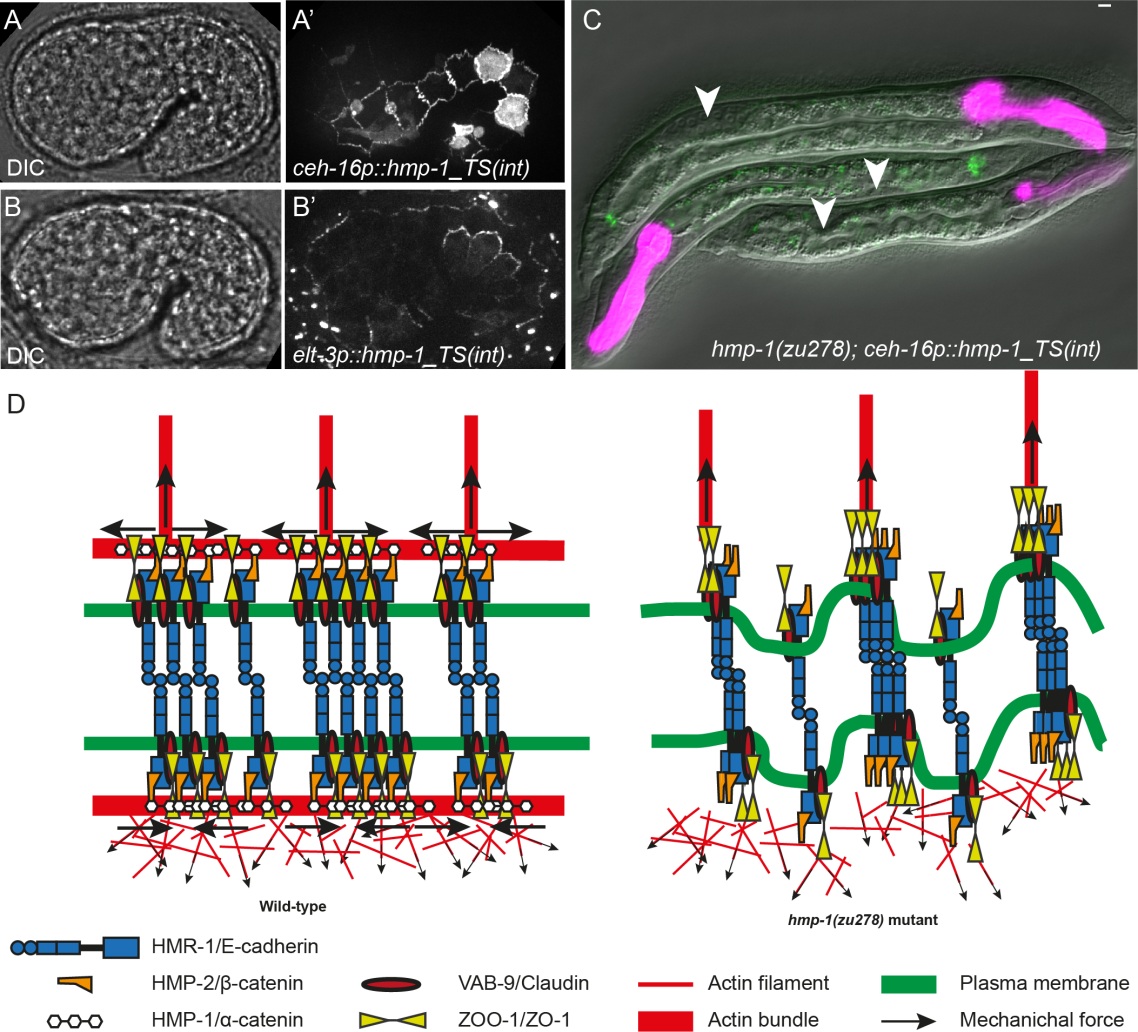
HMP-1 expression either in seam or DV cells rescues the Humpback and embryonic lethal phenotype. (A-A’) DIC and Venus fluorescence pictures of an embryo expressing HMP-1_TS(int) under the epidermal promoter *ceh-16*, showing strong expression in seam cells. Note that some accumulation of HMP-1 between HYP6/HYP7 cell borders is also observed. (B-B’) DIC and Venus fluorescence pictures of an embryo expressing HMP-1_TS(int) under the epidermal promoter *elt-3*, showing strong expression in dorso-ventral cells. (C) *hmp-1(zu278)* larvae rescued with HMP-1 expressed under the *ceh-16* promoter. The arrowheads mark the position of germ-cell precursors; note that rescued larvae have a rather short tail. The pharyngeal magenta marker and junctional green fluorescence indicate the presence of the transgene. Scale bar, 5 μm. (D) Model showing how HMP-1 helps to maintain the local force balance at the junction; dorsal is up. The presence of HMP-1 induces the accumulation of actin at seam-dorso/ventral sides of the junctions (left); the absence of HMP-1 leads to a strong reduction of junctional actin (right; see also S3 Fig) and HMR-1 clustering. Note that the distribution of actin is hypothetical.

To assess the importance of HMP-1 in seam versus in dorso/ventral epidermal cells, we examined whether specific HMP-1 expression in each cell type would rescue the lethality and body morphology defects of the *hmp-1(zu278)* mutant. We used the HMP-1_TS(int) plasmid construct, which we expressed under the seam- and dorso/ventral-cell specific promoters *ceh-16* and *elt-3*, respectively [30, 36–38] (Fig 5A, 5A’, 5B and 5B’). Surprisingly, expression of HMP-1 either in seam or in dorso-ventral epidermal cells partially rescued the embryonic lethality of *hmp-1(zu278)* mutants (S4 Table). The surviving larvae in both cases looked similar: they had a short tail (Fig 5C) and were sterile. HMP-1 expressed under the *nhr-73* promoter, another seam-specific promoter [39], also rescued the embryonic lethality of *hmp-1(zu278)* (S4 Table). The level of rescue was slightly higher when HMP-1 was expressed only in seam cells: 65% and 44% of predicted homozygous *hmp-1* mutant carrying transgenic *ceh-16*::*hmp-1* and *nhr-73p*::*hmp-1* hatched to make larvae, whereas only 37% of the expected transgenic *elt-3*::*hmp-1* homozygous *hmp-1* larvae could hatch. These results suggest that it is sufficient to have HMP-1 in either seam cells or in dorso-ventral cells to achieve the rescue of Humpback phenotype and embryonic lethality. Alternatively, it is possible that, although the promoters we used primarily drive expression in the intended cells, there is some low-level expression in other cells that allows the rescue.

## Discussion

In this work, by using a FRET-based force sensor integrated in the endogenous HMP-1/α-catenin to assess the tension exerted on AJs and by performing time-lapse fluorescence microscopy we revisited the role of HMP-1 during *C*. *elegans* embryonic elongation. We observed a cell-specific distribution and a decrease of tension from the 1.3-fold to 1.5-fold stages. We also found that the regulation HMR-1/E-cadherin turnover does not depend on the force applied to HMP-1/α-catenin. Finally, our characterization of E-cadherin distribution in *hmp-1* mutants suggests that HMP-1’s main function is to maintain adherens junction mechanical integrity, but does not involve the anchoring of actin bundles to HMR-1.

Actomyosin contractility is essential to drive embryonic elongation in *C*. *elegans*. In a recent study, we have shown that tension on the actin cortex increases as the embryo elongates from the 1.3-fold to the 1.7-fold stage [34]. Surprisingly, we found a decrease of tension on the junctions from 1.3-fold to the 1.5-fold stage, correlating with more wrinkled junctions at that later stage (Fig 2C–2F). The absence of correlation between tension applied on junctions through HMP-1 and cortical tension suggests that the first does not necessarily reflect the second, and that myosin II activity is not regulated similarly at the junctions and at the cortex. In agreement with the notion that tension decreases at junctions after the 1.3-fold stage, Piekny *et al*. observed that MEL-11/Myosin Phosphatase Targeting Subunit gets sequestered at junctions [40] from the 1.3-fold to the 1.5-fold stage, which should inhibit contractility at junctions. It also suggests that the tension between epidermal cells can be transmitted through junctional proteins other than HMP-1 or through other components, such as the apical extracellular matrix as we recently demonstrated distributes some of the cortical tension [41]. Consistent with this hypothesis, seam cell in *hmp-1* or *hmp-1; vab-9* mutant embryos were markedly more extended in the DV direction compared to control embryos, suggesting that there was still pulling (tension) from dorso/ventral cells on seam cells (Fig 4K, 4L, 4O and 4P, blue shading)”. Last, as discussed further below, our results strongly suggest that HMP-1 function in attaching circumferential actin bundles to HMR-1 is dispensable, which is again consistent with the notion that it does not bear the brunt of circumferential tension.

Several studies have shown that AJs are mechano-sensitive [15, 42]. However the conclusions from in vitro studies analysing the role of tension on E-cadherin recruitment are rather unclear: some suggest that it depends on myosin II contractility [43, 44] and on external forces applied to junctions [32], whereas others suggest that the change in myosin II contractility affects E-cadherin recruitment through changing actin dynamics rather than a change in tension [45]. Our measurements did not show a correlation between HMR-1 recovery (FRAP measurements) and the tension exerted on HMP-1 (FRET measurements) (Fig 3D and 3E), suggesting that HMR-1 turnover does not depend on actomyosin tension. Several limitations of the FRET approach may account for our failure to detect the mechano-sensitivity of the junctions. Firstly, as shown by the relatively small dynamic range of the FRET index compared to hyper- and hypo-tension (Fig 2G), the sensor that we used may not be sensitive enough within the tension range to which *C*. *elegans* junctions are exposed. Furthermore, as discussed above, the tension on HMP-1 may not reflect the sum of forces applied on adherens junctions, since HMR-1 is connected to actin through other actin binding molecules, such as ZOO-1 (through VAB-9). In addition, some HMP-1 molecules on the junction may not be under tension. Thus, the FRET index observed may represent an average value depending on the proportion of HMP-1 molecules under tension, and a change in this proportion may undervalue a change in the magnitude of actomyosin forces exerted on the junction. Despite these potential limitations, our FRET controls and genetic data (Fig 2B and 2G) support the conclusion that the HMR-1 turnover does not depend on the tension exerted on HMP-1. Interestingly, a recent study described the development of another α-catenin FRET-based conformational sensor in tissue culture cells [46]. The donor and acceptor fluorophores positioned on each side of the central regulatory domain reports the unfolding of α-catenin in response to actomyosin tension. A key aspect of their study lays in the demonstration that tension exerted on α-catenin induces the recruitment of vinculin, an event that does not occur in *C*. *elegans* since vinculin is not expressed in the epidermis. Whether HMP-1 unfolds in response to tension and recruits other molecules remains to be elucidated in the future.

The FRET approach also indicates that the tension exerted on HMP-1 does not correlate with the shortening speed of the seam-seam junctions (DOI: 10.6084/m9.figshare.5878138). In a recent paper, Martin *et al*. suggested that the greater shortening speed of the posterior seam-seam junction of H1 (H1-P) compared to the anterior one (H1-A) was due to higher actomyosin force on H1-P [47]. The authors used the heterogeneity of the junctional marker AJM-1 as an indication of forces applied on the junctions. We also observed a more rapid shortening of H1-P, however our tension measurement did not indicate that higher forces are exerted on H1-P which might reflect the differences in the methods used to measure forces. Since AJM-1 and its partner DLG-1/Discs Large act in a complex contributing to the integrity of epithelial junctions different from AJs [48, 49], it is not known how tension reported by AJM-1 heterogeneity is related to tension on HMP-1. A tension sensor integrated in HMR-1 could be a better solution to compare the total forces applied on AJs.

HMP-1 and VAB-9 have been proposed to mediate actin bundle anchoring to the junctions in dorso-ventral epidermal cells [7, 31]. Instead, while our results support the anchoring role of VAB-9, they suggest that HMP-1 is important to ensure the mechanical integrity and local force equilibrium of seam-dorso/ventral junctions. Indeed, firstly, in embryos with zygotic depletion of HMP-1, HMR-1/E-cadherin detached together with actin bundles that retracted dorsally, indicating that the link between HMR-1 and actin bundles in dorso-ventral cells was still present. Secondly, in *vab-9; hmp-1* double mutants, HMR-1 pulling away from junctions was reduced, implying that the absence of VAB-9 reduces the tension exerted on HMR-1 from the DV epidermis side. Thirdly, using our FRET tension sensor, we did not observe an increase of actomyosin tension on HMP-1 in *vab-9* mutant embryos, suggesting that HMP-1 and VAB-9 perform different functions. Finally, the seam-cell-specific and to a slightly lower level the dorso/ventral-cell-specific expression of HMP-1 both rescued the Humpback phenotype, suggesting that the presence of HMP-1 at seam-DV junctions may be important. Altogether, we suggest that HMP-1 maintains the mechanical integrity and the force equilibrium at the seam/dorso-ventral cell borders by recruiting a specific pool of actin to the junction belt (Fig 5D). Indeed, *hmp-1* mutants show an absence of junctional actin [7]. Furthermore, junctional actin dynamics impacts on elongation [50], supporting the importance of this junction belt. In addition, we also observed irregular actin organization at seam-dorso/ventral borders in *hmp-1* mutant (S3 Fig). Mechanically, the junctional actin belt could distribute the actomyosin forces laterally (along the anterior-posterior direction), as was previously suggested [35]. It may also increase the stiffness of the junctions, to allow equilibrium between a discrete force pattern (DV cells) and a more homogeneous force pattern (seam cells). Finally, the reduction of the actin junctional belt might affect the balance of E-cadherin *cis-*and *trans-*dimers [51], which in turn could make the junction less stable and prone to get pinched within the dorso-ventral cells. The significant elongation rescue of *hmp-1* mutants observed after expressing HMP-1 only on the DV or the seam side (using DV- or seam-cell-specific promoters) suggests that the role of HMP-1 in reinforcing junction stiffness and/or the *cis/trans* clustering of HMR-1 can propagate across the junction. Thereby, HMP-1 assists the resistance of the seam-dorso/ventral junctions to the pulling of actin bundles perpendicular to them and maintains the local force equilibrium at the seam-DV junctions (Fig 5D). The rescue by seam-cell-specific expression of HMP-1 supports the hypothesis that HMP-1 is necessary to recruit junctional actin but dispensable to connecting actin bundles to HMR-1 in dorso/ventral cells.

In summary, using imaging and biophysical approaches, we have provided a quantitative insight in the regulation of junction mechanical integrity and dynamics. Since adherens junctions are implicated in cell physiology and tissue maintenance [3, 52, 53], our studies are not only important to understand morphogenesis but also organism homeostasis and diseases such as cancer.

## Materials and methods

### *C*. *elegans* alleles and strains

We used the Bristol strain N2 as the wild-type strain. Nematodes were cultured in nematode growth medium as described in [54]. The strains used are listed in S5 Table.

### CRISPR knock-in and transgenesis

CRISPR knock-ins were either carried out using a protocol described elsewhere [55, 56] or using a modified protocol using single worm PCR detection (HMP-1_TS(int) and HMP-1_TS-TRAF constructs). The HMP-1_TS-5aa construct was obtained from shortening the 40 aa linker in the HMP-1_TS(int) construct to 5aa, using a single DNA strand containing the deletion as the repair template [57] and single worm PCR detection.

For rescue of *hmp-1(zu278)* phenotype, 2.9 kb and 1.9kb of *ceh-16* and *elt-3* promoter region as described previously in [30] or 1 kb of *nhr-73* promoter region, was put upstream of HMP-1_TS(int) sequence with *unc-54* 3’UTR. The plasmids were injected at 5 ng/μl concentration together with co-injection markers red pharynx (*myo-2p*:*mCherry*) 2.5 ng/μl and roller (*rol-6(su1006)*) 100 ng/μl.

### Immuno-staining to calculate junction length

Embryos were freeze-cracked using liquid nitrogen and fixed on glass slides with cold methanol. The junctions were then stained with MH27 antibody [24].

### FRET image acquisition

Embryos were mounted on a glass slide in M9 solution on a 5% agarose pad. Fluorescent pictures were acquired using a Roper Scientific spinning disk system (Zeiss Axio Observer.Z1 microscope, Yokogawa CSUX1-A1 confocal head, Evolve EMCCD 512x512 pixel camera, Metamorph software) with a 100X oil-immersion objective, NA = 1.4. Donor (mTFP1) and acceptor (Venus) were excited using a 457 nm and a 515 nm diode laser, respectively. Donor and acceptor emission was detected using a 490/40 nm and 545/40 nm bandpass filter, respectively. The power of the two excitation lasers (457 and a 515 nm) was fixed at 50% and 20%, respectively, for all FRET measurements. The exposure time was the same for both donor and acceptor fluorescence, and was tuned to either 150 ms or 400 ms depending on the fluorescence intensity of the transgenic line, to avoid saturation.

The embryos were kept at 20°C during imaging except otherwise mentioned. The acquisition sequence followed three steps:

- FRET fluorescence *I*_*FRET*_ (457 nm excitation, 545/40 nm detection)
- Donor fluorescence *I*_*donor*_ (457 nm excitation, 490/40nm detection)
- Acceptor fluorescence *I*_*acceptor*_ (515nm excitation, 545/40nm detection).

Since the *rga-2(hd102)* embryos are embryonic lethal [25], we imaged embryos from heterozygous mothers then checked the phenotype of the embryos 45 min after acquisition. We only analysed images of the embryos exhibiting the rupture phenotype typical of *rga-2(hd102)* mutants.

### Spectral Bleed Through (SBT) correction and FRET index calculation

Donor Bleed Through (DBT) and Acceptor Bleed Through (ABT) were determined using *hmp-1* CRISPR knock-in lines expressing a C-terminal fusion of mTFP1 only (HMP-1::mTFP1(Cter)) and Venus only (HMP-1::Venus(Cter)), respectively.

All images were analysed using ImageJ (Wayne Rasband, National Institute of Health, USA). SBT and FRET index were computed using the plugin PixFRET [58]. The DBT and ABT were measured the same day or the day after FRET measurements. Their values were derived using the following equations:

$$DBT=\frac{I_{FRET}}{I_{donor}}\;\left(Using\;HMP‑1::mTFP1\left(Cter\right)\;strain\right)$$ (1)

$$ABT=\frac{I_{FRET}}{I_{acceptor}}\;\left(Using\;HMP‑1::Venus\left(Cter\right)\;strain\right)$$ (2)

An average of both SBT was then calculated over at least 10 embryos for each line. The exposure time for images used to calculate SBT was the same as that used to image and calculate FRET index (150 ms or 400 ms).

The FRET index was computed after correction for background noise, measured in the cytoplasm of the seam cell examined or from several seam cells if the FRET index of the whole embryo was calculated. The normalized FRET (NFRET) image was calculated using the following equation:

$$NFRET=\frac{I_{FRET}-DBT\times I_{donor}-ABT\times I_{acceptor}}{I_{donor}}\times 100$$ (3)

We chose only the junction region by manually setting a threshold on the acceptor fluorescence picture and eliminating obvious artefacts outside the junctions. The FRET index of the whole embryo or a particular junction was obtained from averaging NFRET values for the all junctions or the selected junction, respectively.

### Actin distribution imaging with deconvolution (S3 Fig)

Z-stack images of LIFEACT::GFP marker expressed under an epidermal promoter were acquired using a confocal Leica SP5 microscope with a 63X oil-immersion objective and zoom factor 8. A step size of 0.08 μm and a pinhole opening of 0.6 Airy Unit were used. Images within 1 μm on each side of the actin cortex were projected then deconvolved using the Huygens Essential software from Scientific Volume Imaging (Hilversum, Netherlands).

### Junctional roughness measurements

Junctional roughness measurements were performed on acceptor images of HMP-1_TS(int) embryos. We used a homemade Mathlab program (The MathWorks, Inc., Natick, Massachusetts, United States). We traced manually along the junction from one vertex to another to calculate the junction length *l*_*act*_. We also traced a line passing through all the vertices to have the theoretical minimal length of the junction *l*_*min*_. The roughness was then calculated with the following formula:

$$s=\frac{l_{min}}{l_{act}}$$ (4)

For each embryo, we measured 3 seam-seam and 3 seam-dorso/ventral junctions without priotizing a particular cell.

### FRAP imaging and quantification

FRAP measurements were performed on a CRISPR HMR-1::GFP line [33] using the iLaS^2^ module integrated to the same spinning disk system described previously. A disk of 2 μm in diameter centered on the junctions was photo-bleached using 100% power of the 491 nm laser. For each time point, a Z-stack of 5 focal planes, centered on the focal plane of chosen region and spaced by 0.35 μm, was acquired. We took five Z-stacks before photobleaching and imaged up to 150 s (29 Z-stacks with different time interval) after bleaching. To correct for the photobleaching during image acquisition, we continuously acquired 34 Z-stacks of control embryos.

For image analysis, a Z-projection was performed for all the time points using ImageJ. The movement of junctions during the acquisition was corrected using the StackReg plugin [59]. The mean fluorescence intensity was calculated over the bleached region for FRAP experiments.

The correction curve for photobleaching due to image acquisition was calculated as follow:

$$C\left(t_{i}\right)=\frac{I\left(t_{i}\right)}{I\left(t_{1}\right)}$$ (5)

where I(*t*_*i*_) was the mean intensity (after background subtraction) at time point *i* and I(*t*_*1*_) the intensity at the first time point. The corrected fluorescence intensity was determined using the following equation:

$$I_{cor}\left(t_{i}\right)=\frac{I_{bl}\left(t_{i}\right)}{C\left(t_{i}\right)}$$ (6)

where *I*_*bl*_*(t*_*i*_*)* was the mean fluorescence intensity of the bleached region (after background subtraction) at time point *i*. The fluorescence intensity of bleached regions was then normalized as follow:

$$I_{nor}\left(t_{i}\right)=\frac{I_{cor}\left(t_{i}\right)-I_{min}^{post}\left(t_{i}\right)}{I_{mean}^{pre}\left(t_{i}\right)-I_{min}^{post}\left(t_{i}\right)}$$ (7)

where $I_{min}^{post}(t_{i})$ was the minimal corrected intensity after FRAP, and $I_{mean}^{pre}(t_{i})$ was the average corrected intensity before FRAP.

Normalized fluorescence intensity after photobleach was plotted versus time using Matlab and fitted using the equation:

$$y=M\left(1-e^{\frac{-tln2}{\tau }}\right)$$ (8)

where *M* was the mobile fraction and *τ* was the half-time of recovery. The immobile fraction of recovery was calculated as 1-*M*. From the fitting we derived the standard error of the fit which was reported.

### Time-lapse fluorescence movies

Time-lapse movie was carried out using the previously described spinning disk system using at 63x oil-immersion objective, NA = 1.4. Z-stack of the upper part of the embryo (around 5 μm range) with 0.3 μm step size were acquired to show the junctional defects in control, *hmp-1(zu278)* and *vab-9(e1744)* mutant.

The HMR-1 extensions into dorsal cells in *hmp-1(zu278)* and *hmp-1(zu278); vab-9(e1744)* backgrounds were counted from the dorsal side of H2, V1, V2 at the moment when the embryo muscles start to contract. We choose muscle contraction start as a reference time instead of embryo length, because the different genotypes may have different elongation rate.

### Junction fragmentation quantification

To select the junctional area, images of HMR-1::GFP embryos at 1.5-fold stages were thresholded manually using ImageJ, with a threshold based on the cytoplasmic fluorescence level. The junctions of H1 and V3 were selected and split into distinct regions of interest (ROI, Parts A-C in S2 Fig). The number of fragments or ROI for each junction with a minimum size greater than 4 pixels was processed using Matlab.

### Statistical analysis

Mann-Whitney tests were performed using GraphPad Prism.

## Supporting information

S1 Fig FRAP experiment to assess HMR-1/E-cadherin turnover. (A) Scheme showing the FRAP experiment on the posterior junction of the seam cell H1 (H1-P). The yellow circle shows the FRAP area. Symbols for designing junctions are as in Fig 1A”. Timing on the four right pictures is with respect to the first image after-bleach (0 s). Scale bar, 5 μm. (B) Example of normalized average fluorescence recovery data fitted with a single exponential fit (see Materials and Methods). (C and D) FRAP mobile fraction for seam-seam (C) and seam-dorso/ventral (D) junctions in different genetic backgrounds. (E and F) recovery half-time for seam-seam (E) and seam-dorso/ventral (F) junctions in different genetic backgrounds. *WT*, *vab-9*, *hmp-1* denote wild-type, *vab-9(e1744)*, and *hmp-1(zu278)* mutants, respectively. Each data point is obtained from fit of a single embryo recovery curve. Red lines show average and s.e.m; p-values of Mann-Whitney test are reported. ns, not significant; *, p<0.05; **, p<0.01; ***, p<0.001; black p-values and brackets show comparison between the same genotypes; vertical red p-values are calculated for junctional pairwise comparison with WT. Kruskall-Wallis test for all WT junctions gives values p = 0.004 (C), p = 0.44 (D), p = 0.005 (E) and p<0.0001 (F). Number of junctions used for FRAP experiments is: WT H1 (A = 35, P = 35, D = 19, V = 30); WT V1 (A = 36, P = 29, D = 27, V = 30); WT V3 (A = 50, P = 49, D = 56, V = 57); *vab-9(e1744)* H1 (A = 22, P = 35, D = 24, V = 31); *vab-9(e1744)* V1 (A = 35, P = 29, D = 26, V = 29); *vab-9(e1744)* V3 (A = 28, P = 28, D = 29, V = 30); *hmp-1(zu278)* H1 (A = 20, P = 24, D = 23, V = 24); *hmp-1(zu278)* V3 (A = 20, P = 22, D = 20, V = 27).
(TIF)

S2 Fig Junction fragmentation in *hmp-1(zu278)* mutant. (A) Example of an *hmp-1(zu278)* embryo at the 1.5-fold stage showing a fragmentation of the HMR-1::GFP marker most prominent in the head (H1, yellow dashed rectangle, compared to Part A in S1 Fig). Panels below show a close-up view of H1 with the detection of fragments highlighted by yellow contours. (B) Comparison of the number of detected fragments in H1. p-values of Mann-Whitney test are shown; ***, p<0.0001; plus sign shows the mean; WT, wild-type. Number of junctions used for quantification: WT H1 (A = 25, P = 30, D = 29, V = 23), *hmp-1(zu278)* H1 (A = 11, P = 15, D = 24, V = 15).
(TIF)

S3 Fig Junctional actin is disorganized in *hmp-1* mutants. A wild-type (A) and a *hmp-1(zu278)* mutant (B) late elongation embryo expressing the actin reporter LIFEACT::GFP showing disorganized seam-dorso/ventral epidermal cell borders (yellow dashed contours) in the mutant. Arrows showing humps on the back of the embryo. Actin images were acquired using a scanning confocal microscope followed by deconvolution as described in Materials and Methods. Scale bar, 5 μm.
(TIF)

S1 Table Embryonic lethality of different HMP-1 lines containing tension sensor or related control constructs. Notation is the same as in the Fig 2A.
(DOCX)

S2 Table Number of embryos used to measure FRET index for different tension sensor constructs. (DOCX)

S3 Table Number of junctions used to measure FRET index in H1, V1 and V3 seam cells of embryos at the 1.5-fold stage. (DOCX)

S4 Table Rescue capability of HMP-1 expressed under different epidermal promoters (*ceh-16*, *elt-3* and *nhr-73*). (DOCX)

S5 Table Strains used in this work. (DOCX)
