# Correction: HMP-1/α-catenin promotes junctional mechanical integrity during morphogenesis

**DOI:** 10.1371/journal.pone.0195419

**Published:** 2018-03-29

**Authors:** The PLOS ONE Staff

The following information is missing from the Funding section: Some confocal work was carried out at the Institute of Biology Paris Seine Imaging facility which is significantly supported by the ‘Conseil Regional Ile-de-France’, the French national research council (CNRS) and Sorbonne University, UPMC Univ Paris 06.

The publisher apologizes for the error.
