# Supplementary figures and images for: HMP-1/α-catenin promotes junctional mechanical integrity during morphogenesis
Source: PLoS One. 2018 Feb 21;13(2):e0193279. doi: 10.1371/journal.pone.0193279 (PMC5821396; doi:10.1371/journal.pone.0193279)

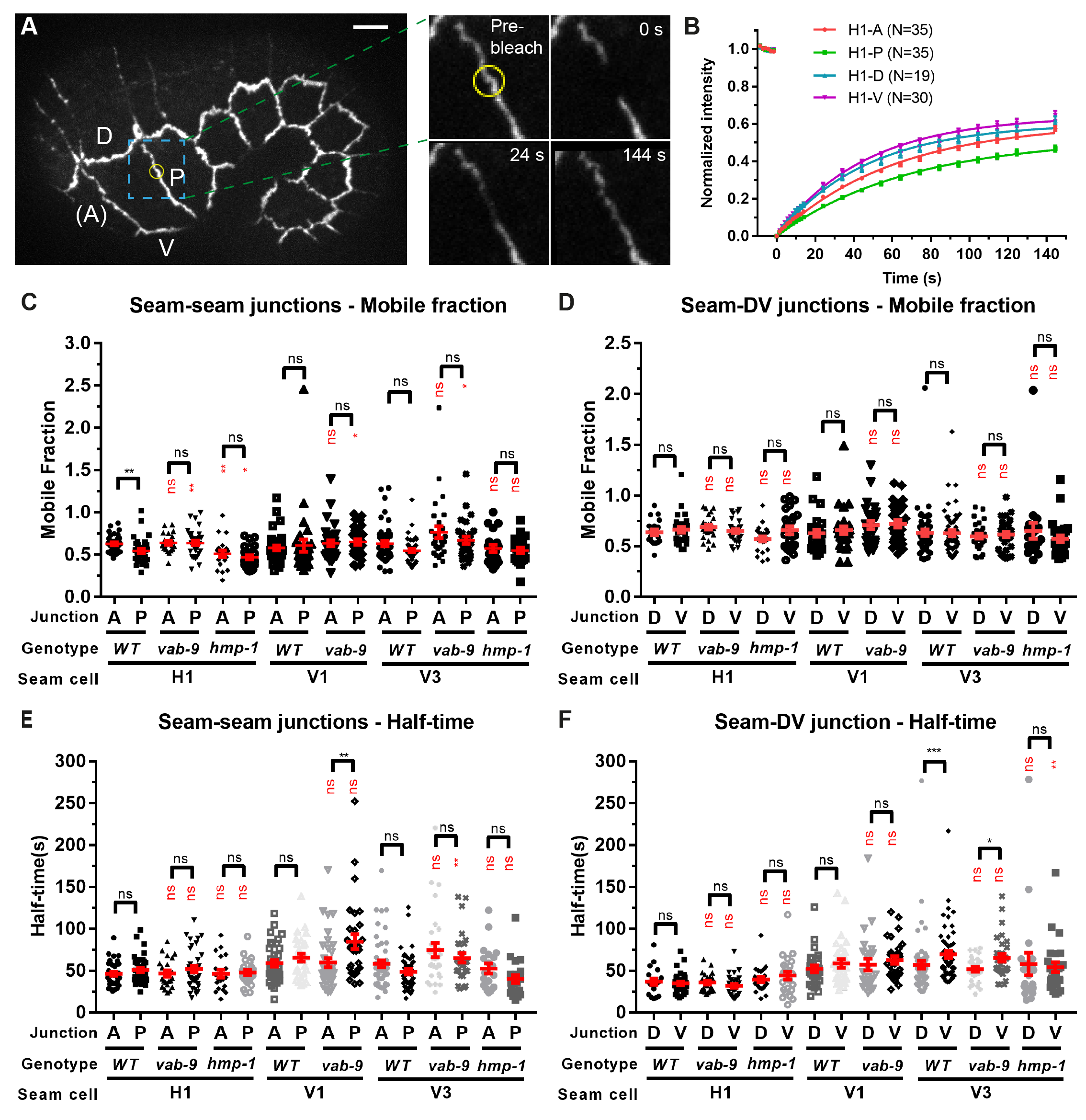

Supplement: S1 Fig — (A) Scheme showing the FRAP experiment on the posterior junction of the seam cell H1 (H1-P). The yellow circle shows the FRAP area. Symbols for designing junctions are as in Fig 1A”. Timing on the four right pictures is with respect to the first image after-bleach (0 s). Scale bar, 5 μm. (B) Example of normalized average fluorescence recovery data fitted with a single exponential fit (see Materials and Methods). (C and D) FRAP mobile fraction for seam-seam (C) and seam-dorso/ventral (D) junctions in different genetic backgrounds. (E and F) recovery half-time for seam-seam (E) and seam-dorso/ventral (F) junctions in different genetic backgrounds. WT, vab-9, hmp-1 denote wild-type, vab-9(e1744), and hmp-1(zu278) mutants, respectively. Each data point is obtained from fit of a single embryo recovery curve. Red lines show average and s.e.m; p-values of Mann-Whitney test are reported. ns, not significant; *, p<0.05; **, p<0.01; ***, p<0.001; black p-values and brackets show comparison between the same genotypes; vertical red p-values are calculated for junctional pairwise comparison with WT. Kruskall-Wallis test for all WT junctions gives values p = 0.004 (C), p = 0.44 (D), p = 0.005 (E) and p<0.0001 (F). Number of junctions used for FRAP experiments is: WT H1 (A = 35, P = 35, D = 19, V = 30); WT V1 (A = 36, P = 29, D = 27, V = 30); WT V3 (A = 50, P = 49, D = 56, V = 57); vab-9(e1744) H1 (A = 22, P = 35, D = 24, V = 31); vab-9(e1744) V1 (A = 35, P = 29, D = 26, V = 29); vab-9(e1744) V3 (A = 28, P = 28, D = 29, V = 30); hmp-1(zu278) H1 (A = 20, P = 24, D = 23, V = 24); hmp-1(zu278) V3 (A = 20, P = 22, D = 20, V = 27). (TIF) [file pone.0193279.s001.tif]

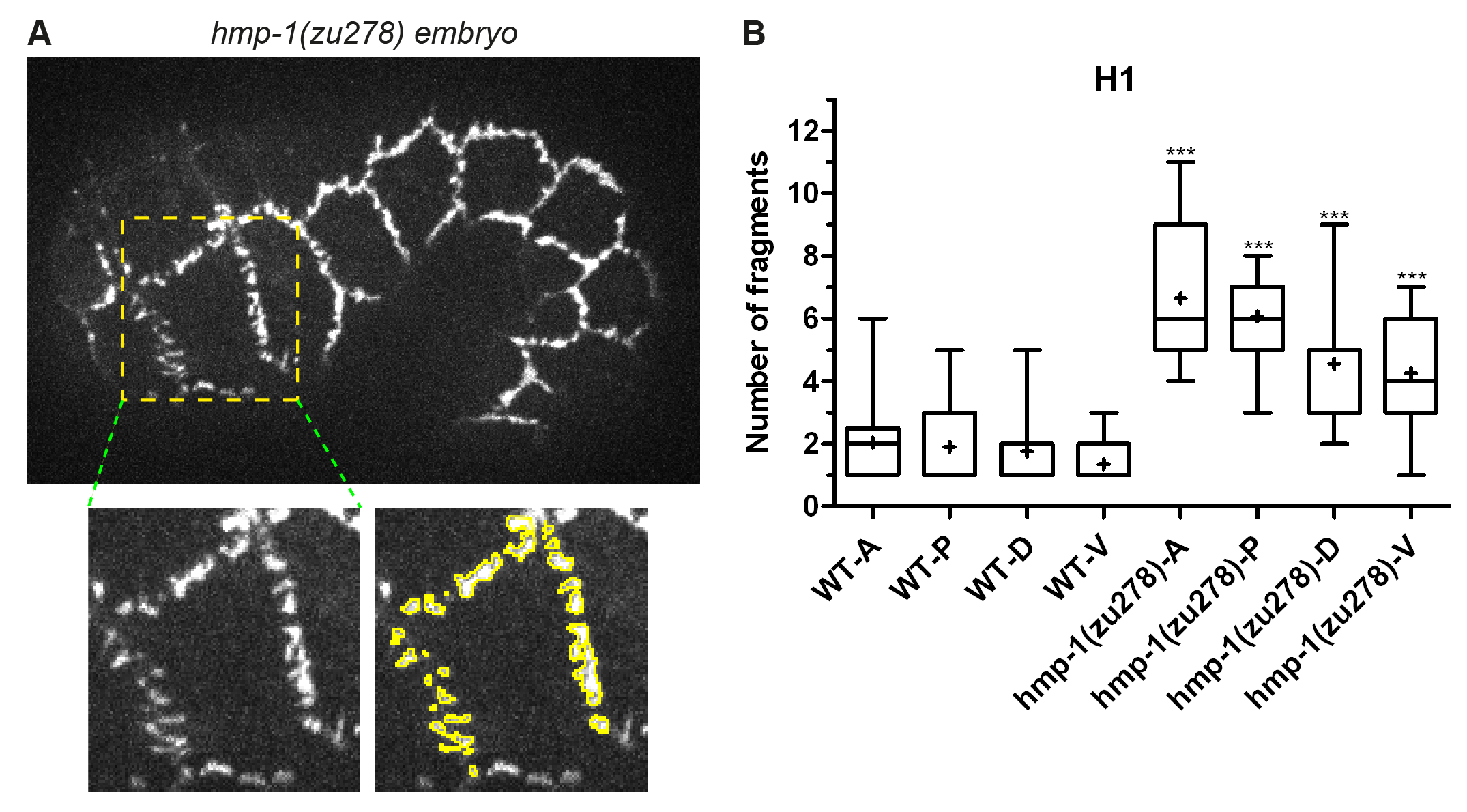

Supplement: S2 Fig — (A) Example of an hmp-1(zu278) embryo at the 1.5-fold stage showing a fragmentation of the HMR-1::GFP marker most prominent in the head (H1, yellow dashed rectangle, compared to Part A in S1 Fig). Panels below show a close-up view of H1 with the detection of fragments highlighted by yellow contours. (B) Comparison of the number of detected fragments in H1. p-values of Mann-Whitney test are shown; ***, p<0.0001; plus sign shows the mean; WT, wild-type. Number of junctions used for quantification: WT H1 (A = 25, P = 30, D = 29, V = 23), hmp-1(zu278) H1 (A = 11, P = 15, D = 24, V = 15). (TIF) [file pone.0193279.s002.tif]

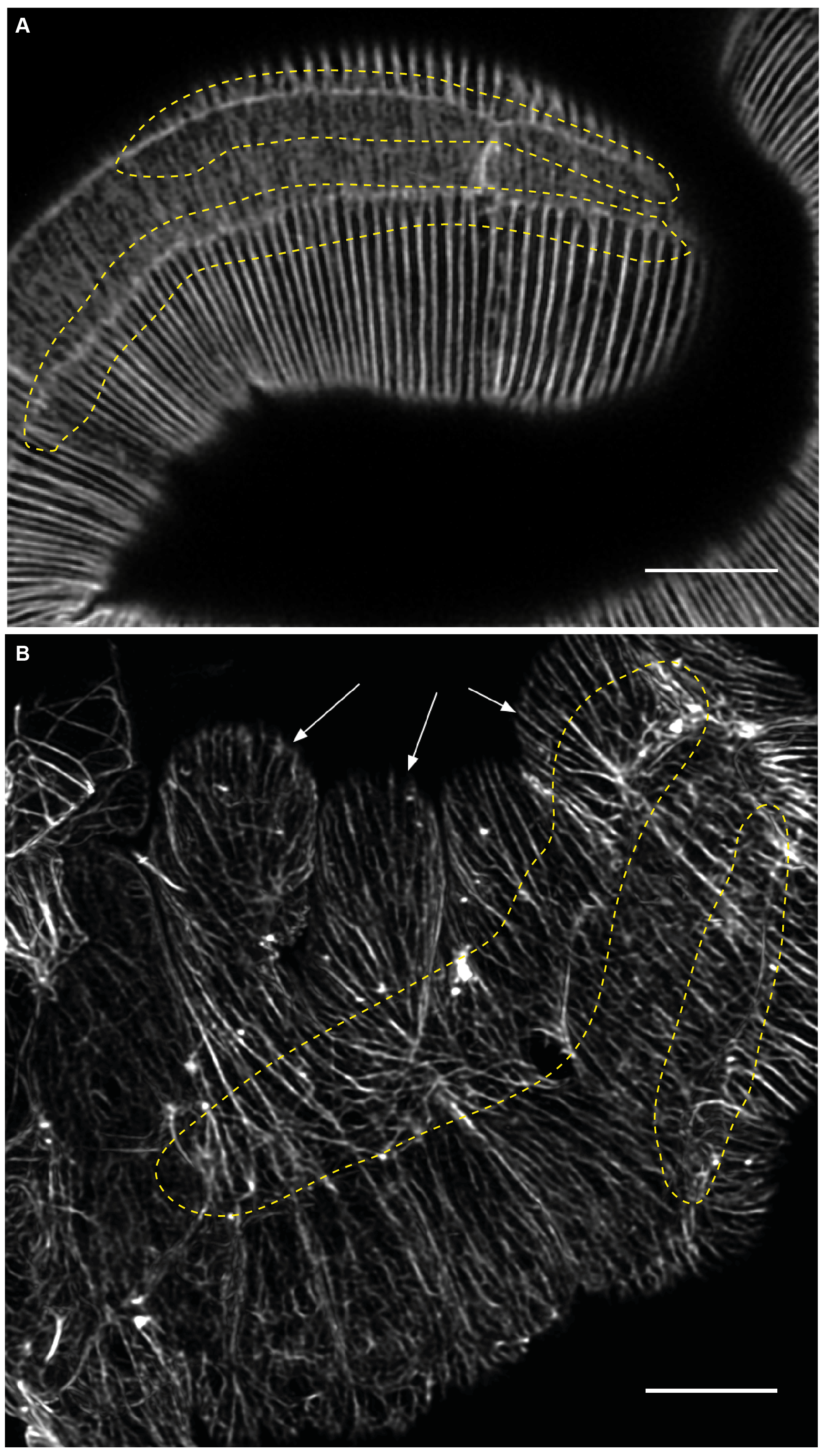

Supplement: S3 Fig — A wild-type (A) and a hmp-1(zu278) mutant (B) late elongation embryo expressing the actin reporter LIFEACT::GFP showing disorganized seam-dorso/ventral epidermal cell borders (yellow dashed contours) in the mutant. Arrows showing humps on the back of the embryo. Actin images were acquired using a scanning confocal microscope followed by deconvolution as described in Materials and Methods. Scale bar, 5 μm. (TIF) [file pone.0193279.s003.tif]
