# Supplementary material for: HMP-1/α-catenin promotes junctional mechanical integrity during morphogenesis
Source: PLoS One. 2018 Feb 21;13(2):e0193279. doi: 10.1371/journal.pone.0193279 (PMC5821396; doi:10.1371/journal.pone.0193279)
Supplement: S1 Table — Notation is the same as in the Fig 2A. (DOCX) [file pone.0193279.s004.docx]

**S1 Table**

| CRISPR contruct | N | % embryonic lethality |
| --- | --- | --- |
| *HMP-1_TS(int)* | 776 | 0.39 |
| *HMP-1_TS-5aa* | 566 | 0.71 |
| *HMP-1::TS(Cter)* | 495 | 3.64 |
