# Supplementary material for: HMP-1/α-catenin promotes junctional mechanical integrity during morphogenesis
Source: PLoS One. 2018 Feb 21;13(2):e0193279. doi: 10.1371/journal.pone.0193279 (PMC5821396; doi:10.1371/journal.pone.0193279)
Supplement: S2 Table — (DOCX) [file pone.0193279.s005.docx]

**S2 Table**

| Line | N |
| --- | --- |
| *HMP-1_TS-5aa* 1.3F | 25 |
| *HMP-1_TS-5aa* 1.5F | 31 |
| *HMP-1_TS(int)* 1.3F | 86 |
| *HMP-1_TS(int)* 1.5F | 57 |
| *HMP-1_TS(Cter)* 1.3F | 46 |
| *HMP-1_TS(Cter)* 1.5F | 27 |
| *HMP-1_TS-TRAF* 1.3F | 35 |
| *HMP-1_TS-TRAF* 1.5F | 24 |
| *HMP-1_TS(int); rga-2(hd102)* 1.5F | 33 |
| *HMP-1_TS(int)* 25.5°C 1.5F | 26 |
| *HMP-1_TS(int); let-502(sb118)* 25.5°C 1.5F | 26 |
