# Supplementary material for: HMP-1/α-catenin promotes junctional mechanical integrity during morphogenesis
Source: PLoS One. 2018 Feb 21;13(2):e0193279. doi: 10.1371/journal.pone.0193279 (PMC5821396; doi:10.1371/journal.pone.0193279)
Supplement: S3 Table — (DOCX) [file pone.0193279.s006.docx]

**S3 Table**

|  | H1-A | H1-P | H1-D | H1-V | V1-A | V1-P | V1-D | V1-V | V3-A | V3-P | V3-D | V3-V |
| --- | --- | --- | --- | --- | --- | --- | --- | --- | --- | --- | --- | --- |
| wild-type | 55 | 63 | 39 | 42 | 52 | 45 | 32 | 32 | 41 | 37 | 30 | 41 |
| *vab-9(e1744)* | 57 | 65 | 39 | 47 | 99 | 68 | 52 | 46 | 40 | 35 | 18 | 32 |
