# Supplementary material for: HMP-1/α-catenin promotes junctional mechanical integrity during morphogenesis
Source: PLoS One. 2018 Feb 21;13(2):e0193279. doi: 10.1371/journal.pone.0193279 (PMC5821396; doi:10.1371/journal.pone.0193279)
Supplement: S4 Table — (DOCX) [file pone.0193279.s007.docx]

**S4 Table**

|  |  | Progeny | | Non-green larvae^3^ | | |
| --- | --- | --- | --- | --- | --- | --- |
| Transgene^1^ | Transgene transmission rate (%) | Total Egg Count | Expected homozygous  *hmp-1*^2^ | Normal body morphology | Red larvae^4^ short tail | % rescue^6^ |
| Control no transgene | NA^5^ | 1020 | 64 | 2 | NA^5^ | NA^5^ |
| *ceh-16p::HMP-1* | 82 | 955 | 60 | 1 | 32 | 65 |
| *elt-3p::HMP-1* | 47 | 912 | 57 | 3 | 10 | 37 |
| *nhr-73p::HMP-1* | 67 | 1133 | 71 | 0 | 21 | 44 |

^1^ All mothers had the genotype *hmp-1(zu278)/nT1[qIs51] (IV;V)* and could carry the transgene indicated in column 1. The genes *ceh-16* and *nhr-73* are specifically expressed in seam cells; *elt-3* is specifically expressed in dorso-ventral epidermal cells. The presence of the balancer *nT1[qIs51]* was scored by checking for green fluorescence in the pharynx; the presence of the extrachromosomal array and transgene was scored by checking for expression of the co-injection marker mCherry under the pharyngeal *myo-2* promoter (red pharynx).

^2^ The fraction of *hmp-1(zu278)* homozygous embryos is 1/16 (6.25%) of the total eggs laid by *hmp-1(zu278)/nT1*[qIs51] *(IV;V)* hermaphrodites due to the segregation of the translocation *nT1*.

^3^ Larvae without the balancer do not have a green pharynx; among them some with a short tail were different from rare escapers of the *hmp-1(zu278)/nT1[qIs51](IV;V)* strain which have normal body morphology.

^4^ Larvae without the balancer having a short tail all carried the transgene (red pharynx).

^5^ NA, not applicable.

^6^The percentage of rescue is calculated as the ratio of rescued larvae (red non-green pharynx with short tail) over the expected number of homozygous *hmp-1* larvae carrying the transgene. The latter is calculated as the number of expected homozygous *hmp-1* larvae times the transgene transmission rate.
