# Supplementary material for: HMP-1/α-catenin promotes junctional mechanical integrity during morphogenesis
Source: PLoS One. 2018 Feb 21;13(2):e0193279. doi: 10.1371/journal.pone.0193279 (PMC5821396; doi:10.1371/journal.pone.0193279)
Supplement: S5 Table — (DOCX) [file pone.0193279.s008.docx]

**S5 Table.**

| Strain name | Genotype |
| --- | --- |
| LP172^a^ | *hmr-1(cp21[hmr-1::GFP + LoxP]) I.* |
| ML1617 | *xnIs97 [hmr-1::GFP]* |
| ML1967 | *mcIs68 [dpy-7p::LifeAct::GFP; unc-119(+)] II* |
| ML2347 | *hmp-1(mc54[hmp-1_TS(int)]) V* |
| ML2355 | *let-502(sb118ts) I ; hmp-1 (mc54[hmp-1_TS(int)]) V* |
| ML2383 | *unc-119(ed3) III; hmp-1(mc59[hmp-1::Venus, loxP::unc-119(+)::loxP]) V* |
| ML2386 | *mcIs50 [lin-26p::ABD::GFP; myo-2p::GFP] I; xnIs97[hmr-1::GFP] III* |
| ML2387 | *unc-119(ed3) III; hmp-1(mc60[hmp-1::mTFP-1; loxP::unc-119(+)::loxP]) V* |
| ML2414 | *unc-119(ed3) III ; hmp-1(mc63[hmp-1::TS(Cter); loxP::unc-119(+)::loxP]) V* |
| ML2629 | *rga-2(hd102)/hIn1 [unc-54] I; hmp-1(mc54[hmp-1_TS(int)]) V* |
| ML2632 | *hmp-1(mc105[hmp-1_TS-TRAF])/daf-11(m84) sma-1(e30) V* |
| ML2633 | *hmp-1(mc106[hmp-1_TS-5aa]) V* |
| ML2676 | *hmr-1(cp21[hmr-1::gfp+LoxP] I; mcIs43[lin-26p:ABD::mCherry, myo-2p::GFP] IV* |
| ML2687 | *hmr-1(cp21[hmr-1::gfp+LoxP] I; hmp-1(zu278)/daf-11(m84) sma-1(e30) V;* |
| ML2686 | *hmr-1(cp21[hmr-1::gfp+LoxP] I; mcIs43[lin-26p:ABD::mcherry, myo-2p::GFP] IV; hmp-1(zu278)/daf-11(m84) sma-1(e30) V;* |
| ML2748 | *mcIs68 [dpy-7p::LifeAct::GFP; unc-119(+)] II; hmp-1(zu278)/nT1(IV;V);* |
| ML2753 | *hmr-1(cp21[hmr-1::gfp + LoxP]) I; vab-9(e1744) II; mcIs43[lin-26p::ABD::mcherry, myo-2p::GFP] IV* |
| ML2755 | *hmr-1(cp21[hmr-1::gfp + LoxP]) I; vab-9(e1744) II; mcIs43[lin-26p:ABD::mcherry, myo-2p::GFP] IV; hmp-1(zu278)/+ V* |
| ML2757 | *hmp-1(zu278) V/nT1[qIs51] (IV;V); mcEx953[ceh-16p::hmp-1_TS(int), myo-2p::mCherry, rol-6(su1006)]* |
| ML2759 | *hmp-1(zu278) V/nT1 [qIs51] (IV;V); mcEx955[elt-3p::hmp-1_TS(int), myo-2p::mCherry, rol-6(su1006)]* |
| ML2770 | *vab-9(e1744) II; hmp-1(mc54[hmp-1_TS(int)]) V* |
| ML2787 | *hmp-1(zu278) V/nT1 [qIs51] (IV;V); mcEx962[nhr-73p::hmp-1_TS(int), myo-2p::mCherry, rol-6(su1006)]* |
| ML2833 | *hmp-1(zu278) V/ nT1 [qIs51] (IV;V)* |

^a^ Strain obtained thanks to the courtesy of Bob Goldstein
